# Assessing the Effectiveness of an Awareness Program on Knowledge and Practice Regarding the Mothers’ Absolute Affection Program Among Postnatal Mothers in Selected Hospitals: Protocol for a Quasi-Experimental Study

**DOI:** 10.2196/73496

**Published:** 2026-03-30

**Authors:** Vaishnavi Dongare, Kavita Gomase, Vaishali Taksande

**Affiliations:** 1 Smt Radhikabai Meghe Memorial College of Nursing, Datta Meghe Institute of Higher Education and Research, Sawangi (Meghe), Wardha, Maharashtra, 442001, India, 91 7397924416

**Keywords:** maternal health, health education, infant feeding practices, breastfeeding promotion, behavioral change communication

## Abstract

**Background:**

The Mothers’ Absolute Affection (MAA) program is a national initiative launched by the Ministry of Health and Family Welfare, Government of India, on August 5, 2016, to promote, support, and protect breastfeeding across the country. The program aims to provide focused breastfeeding promotion and counseling services through the health system. It targets approximately 39 million pregnant and nursing mothers, 880,000 Accredited Social Health Activists, 150,000 subcenters, and 17,000 delivery points across all states and union territories. Despite its wide reach, there remains a lack of valid and reliable tools to assess mothers’ knowledge and practices related to breastfeeding and the MAA program.

**Objective:**

The aims of this study are to assess the existing knowledge and practice regarding the MAA program among postnatal mothers, to evaluate the effectiveness of an awareness program on knowledge and practice regarding the MAA program, to correlate knowledge and practice regarding the MAA program among postnatal mothers, and to associate knowledge and practice with selected demographic variables.

**Methods:**

A quasi-experimental one-group pretest and posttest design was used to evaluate the effectiveness of an awareness program for the MAA program among postnatal mothers. The study was conducted at a selected hospital with a sample of 80 postnatal mothers selected through inclusion and exclusion criteria. On Day 1, participants underwent a pretest followed by an awareness session on the MAA program. A posttest was administered on Day 2. Knowledge was assessed using a structured questionnaire consisting of 15 items, while practice was evaluated using a 5-point Likert scale. Data were analyzed using descriptive and inferential statistics, including paired *t* tests and *χ*^2^ tests.

**Results:**

Knowledge scores were classified into three levels—poor (0%‐33.33%), fair (33.34%‐66.66%), and good (66.67%‐100%)—based on the number of correct responses out of 15. Practice was categorized as poor (0%‐50%) or good (51%‐100%) based on the 5-point scale score. The study revealed significant improvements in both knowledge and practice following the awareness intervention, indicating that the program was effective. The scoring procedure provided a comprehensive understanding of the awareness and practical implementation of the MAA program among postnatal mothers.

**Conclusions:**

The awareness program significantly improved the knowledge and practices of postnatal mothers regarding the MAA program, emphasizing the value of educational interventions in promoting breastfeeding.

## Introduction

### Background

The Ministry of Health and Family Welfare of India developed an enhanced initiative that aims to add to ongoing health system initiatives by placing a clear emphasis on breastfeeding promotion. The program is known as the Mothers’ Absolute Affection (MAA) program. After its national launch in August 2016, states and union territories were able to implement it [1]. The MAA program is a national initiative that aims to bring unwavering attention to breastfeeding promotion and offer counseling services to support breastfeeding through health systems [2].

Breastfeeding is a crucial intervention for the survival of children. Thirty percent of newborn deaths may be avoided if breastfeeding begins within an hour of delivery. Children who are not breastfed have a 15-fold increased risk of pneumonia and a 10-fold increased risk of diarrhea compared to those who receive only breast milk. Efforts to instruct mothers on the most effective breastfeeding techniques must be intensified in light of the overwhelming evidence that breastfeeding reduces infant and neonatal mortality. To enhance feeding for infants and young children, Tamil Nadu has thus been implementing the MAA program since August 2016. Breastfeeding is the closest thing to a miracle cure for child survival [3]. To increase breastfeeding rates, the MAA program seeks to reinvigorate efforts to promote, protect, and support breastfeeding methods through health systems [4]. The program aims to encourage the best breastfeeding practices and create an environment that is supportive of breastfeeding by focusing on awareness-raising efforts for family members, society, and expectant and nursing mothers. The program aims to promote breastfeeding as a crucial intervention for the survival and development of young children [5], strengthen the lactation support services offered by qualified community health workers and health care professionals at public health facilities, and reward and acknowledge health care facilities with high breastfeeding rates and lactation control procedures in place [6]. Breastfeeding has many benefits for the baby: early skin-to-skin contact keeps the infant warm, it facilitates the early production of breast milk, and colostrum (also known as the “first milk”) helps shield a newborn from illness. In addition, it aids in the development of a close and loving relationship between mother and child, reduces the chances of infants contracting diseases like pneumonia or ear and throat infections, is associated with increased IQ, and guarantees growth and development. There are also benefits for the mother as breastfeeding facilitates placental expulsion and womb contraction; reduces the likelihood of severe bleeding following delivery; lowers the chance of developing ovarian, uterine, and breast cancer; diminishes osteoporosis; increases birth spacing due to lactational amenorrhea; and encourages weight loss following childbirth. In addition, breastfeeding is less expensive than feeding with formula [7].

### Study Aim, Objectives, and Hypotheses

The primary aim of this study is to assess the effectiveness of an awareness program on the knowledge and practice regarding the MAA program among postnatal mothers in selected hospitals. Specifically, the study seeks to evaluate the existing knowledge and practice levels of postnatal mothers regarding the MAA program, measure the impact of the awareness intervention on improving these parameters, explore the correlation between knowledge and practice, and determine associations with selected demographic variables. To test these objectives, the study proposes several hypotheses. H0_1_ states that there is no significant difference between preintervention and postintervention knowledge and practice regarding the MAA program, while H1_1_ posits a significant difference between preintervention and postintervention knowledge and practice. Similarly, H1_2_ suggests a significant correlation between knowledge and practice, whereas H0_2_ proposes no significant correlation between knowledge and practice. This integrated approach is intended to provide a comprehensive understanding of how structured educational interventions can influence maternal behavior and support breastfeeding promotion through the MAA program.

## Methods

### Study Overview

This quasi-experimental study will be conducted in selected hospitals. The first part of the study (Day 1) involves a researcher providing an explanation of the MAA program to the mother. The posttest will be administered on Day 2. Mothers who participated in this study demonstrated improved knowledge as a result of the awareness program. This study will address the existing gap in MAA-related research by evaluating the effectiveness of structured awareness programs on improving knowledge and practices regarding the MAA program among postnatal mothers, a component that has been underexplored in previous studies [8].

### Criteria for Sample Selection

Inclusion criteria for the study were as follows:

- Postnatal mothers who were present at the time of data collection.
- Postnatal mothers who were willing to participate in the study.

Exclusion criteria were as follows:

- Postnatal mothers who are health workers.
- Postnatal mothers who already attended an awareness program for the MAA program.
- Postnatal mothers who had been diagnosed with a postnatal psychological condition.

### Sample Calculation

The formula comparing paired before and after proportions is presented here:

Primary variable: knowledge regarding breastfeeding practices = 18%

Knowledge regarding breastfeeding practices before the intervention = 18% = 0.18 (as per reference article [9])

Knowledge regarding breastfeeding practices after the intervention = 38% = 0.38 (20% higher)

*Z* (table value at *α*=.05) = 1.96

*Z* (table value at 1*-β*=.80) = 0.84

N = 78 participants (rounded to 80 to account for attrition)

Total sample required = 78

Actual sample size = 80

### Intervention

On the first day, postnatal mothers received a pretest and awareness program about the MAA program. On the second day, the postnatal mother received a posttest to evaluate the awareness program’s efficacy.

The impact of the awareness-raising initiative on MAA program knowledge will be evaluated in a forthcoming study. Knowledge regarding the MAA program was categorized into one of three levels based on the number of correct answers. A total of 15 questions were included. The practice level related to the MAA program was assessed on a 5-point scale. The scoring aimed to capture the frequency and consistency with which mothers followed the key components of the MAA program, thus providing insight into both awareness and real-world application among the participants.

The research results will be released in a journal with indexing. The study began in February 2024.

### Statistical Analysis

The data collected for the study were analyzed using Microsoft Excel (version 10; Microsoft Corp). Initially, descriptive statistics such as frequencies, percentages, means (X̄), and standard deviations (SD) were used to summarize participants’ knowledge and practice scores regarding the MAA program. Inferential statistical tests were then applied to assess the effectiveness of the awareness program. A paired *t* test was conducted to compare pretest and posttest scores within the same group. Additionally, *χ*^2^ tests were used to examine the association between participants’ demographic variables and their knowledge and practice levels. This comprehensive approach allowed for an in-depth understanding of the impact of the intervention.

### Reliability of the Tool

Reliability indicates the degree of consistency, and it is major criteria for assessing the quality and accuracy of tools. In this study, reliability was tested using the Guttman split-half method (knowledge: reliability=0.8948, *r*=0.913; practice: reliability=0.837, *r*=0.916).

### Validity

To determine content validity, the tool was given to 10 experts within the Obstetrics and Gynecology Nursing Department, Smt. Radhikabai Meghe Memorial College of Nursing. Following consultation with the experts, some modifications were made in the framing (ie, wording) of questionnaire items and the same modifications were included in the study.

### Ethical Considerations

The study protocol was reviewed and approved by the institutional ethics committee of Smt. Radhikabai Meghe Memorial College of Nursing, Datta Meghe Institute of Higher Education and Research (deemed-to-be-university), Wardha, Maharashtra, India (approval number ECR/440/Inst/MH/2013/RR-2019; February 29, 2024). A framework for participant consent will be provided for the research, which is based on the hospital. Written informed consent was obtained from all postnatal mothers prior to participation in the study. Participation was entirely voluntary, and participants were informed of their right to withdraw from the study at any time without any effect on the care they received. To ensure participant privacy and confidentiality, no personally identifiable information was collected. All data were deidentified and coded prior to analysis. The collected data were stored securely and were accessible only to the research team. The findings are reported in aggregate form to prevent individual identification. No monetary or nonmonetary compensation was provided to participants for their involvement in the study.

## Results

The study was conducted between February 2024 and April 2024. Data analysis was completed in May 2024. The final manuscript was submitted for publication in March 2025.

The study showed that, after an awareness intervention, postpartum mothers’ knowledge and practice levels of the MAA program significantly improved. A systematic questionnaire consisting of 15 items was used to evaluate knowledge, and the number of correct answers was used to classify the responses as poor (0%‐33.33%), fair (33.34%‐66.66%), or good (66.67%‐100%). Before the intervention, a sizable portion of participants had poor to fair knowledge. However, the number of participants receiving high knowledge ratings significantly increased following the awareness session. Similarly, practice was divided into two categories—poor (0%‐50%) and good (51%‐100%)—based on a 5-point Likert scale. Better adherence to best practices for breastfeeding was indicated by a significant increase in good practice scores in the postintervention data. Chi-square tests demonstrated correlations between demographic factors and results, while paired *t* tests verified that these changes were statistically significant. These results demonstrate how a well-structured educational program can work to raise awareness and improve the practical application of breastfeeding promotion techniques under the MAA program.

## Discussion

### Principal Findings

A similar study conducted in a rural setting found that an educational intervention focused on maternal care significantly increased knowledge among postnatal mothers. The results showed that after the intervention, 75% of mothers had increased knowledge, which aligns closely with the findings of another study [8]. Another study evaluated the effectiveness of a breastfeeding program among postnatal mothers and found significant improvements in both knowledge and practice [10]. The mean knowledge score in their posttest increased from 6.5 (SD 2.0) to 11.2 (SD 1.5), and the practice scores improved by 30%, which is consistent with the findings of this study on the effectiveness of the awareness intervention program regarding the MAA program [11].

Hospitals should encourage breastfeeding because a mother’s milk supply may be insufficient if she does not nurse her infant during the initial days following birth. Furthermore, many mothers stop nursing if they believe their milk supply is insufficient. This leads to them adding something other than breast milk to an infant’s diet, which may expose the young child to diseases. One limitation of this study is that the promotion of a breastfeeding program at the district hospital level does not reach babies born in other clinics or private hospitals. However, it is realistic to say that, with the limited resources at hand, creating and implementing a policy to support breastfeeding at every private hospital would not be possible. In addition to awareness and knowledge, breastfeeding practices are influenced by a range of external factors such as family support, cultural beliefs, workplace environment, and access to lactation counseling. Future interventions should incorporate strategies to address these contextual elements. For instance, involving family members in educational programs or offering follow-up support through community health workers may enhance the effectiveness of awareness efforts [10].

### Conclusion

The study is expected to demonstrate clear distinctions between theoretical knowledge and practical application of the MAA program among postnatal mothers. It will likely emphasize the importance of integrating awareness programs into hospital settings to strengthen both knowledge and practice. Participants are anticipated to demonstrate an improved understanding of the technical aspects of the MAA program, while social elements—such as maternal cooperation and support dynamics—may still present areas for further enhancement. Future research and program planning will aim to bridge these gaps by incorporating more comprehensive components that address both clinical education and sociobehavioral support for breastfeeding practices.

## Supplementary material

10.2196/73496 Checklist 1 SPIRIT checklist.

## Abbreviations

MAA: Mothers' Absolute Affection
SPIRIT: Standard Protocol Items: Recommendations for Interventional Trials
